# Comparison of Endoscopic Submucosal Dissection Application on Mucosal Tumor and Subepithelial Tumor in stomach

**DOI:** 10.7150/jca.47653

**Published:** 2021-01-01

**Authors:** Wen-Hung Hsu, Tzung-Shiun Wu, Meng-Shu Hsieh, Yu-Min Kung, Yao-Kuang Wang, Jeng-Yih Wu, Fang-Jung Yu, Chao-Hung Kuo, Yu-Chung Su, Jaw-Yuan Wang, Deng-Chyang Wu, Huang-Ming Hu

**Affiliations:** 1 Division of Gastroenterology, Department of Internal Medicine, Kaohsiung Medical University Hospital, Kaohsiung, Taiwan.; 2 Department of Medicine, College of Medicine, Kaohsiung Medical University, Kaohsiung, Taiwan.; 3 Department of Internal Medicine, Kaohsiung Municipal Ta-Tung Hospital, Kaohsiung, Taiwan.; 4 Department of Internal Medicine, Kaohsiung Municipal Siaogang Hospital, Kaohsiung, Taiwan.; 5 Division of Colorectal Surgery, Department of Surgery, Kaohsiung Medical University Hospital, Kaohsiung, Taiwan.

**Keywords:** endoscopic submucosal dissection (ESD), gastric subepithelial tumor (SET)

## Abstract

**Background:** Endoscopic submucosal dissection is minimal invasive endoscopic procedure to deal with gastric tumor. Initially, it was developed to resect mucosal neoplasm since 2000 and extended its application to submucosal tumor in the following years. Although the basic ESD skills are similar in gastric mucosal tumor and subepithelial tumor, the success rate, complication may be different between the two types of gastric tumor resection. This retrospective study is conducted to analyze the ESD procedure in gastric mucosal tumor and subepithelial tumor.

**Methods:** From 2007 to 2016, we reviewed all patients who underwent endoscopic submucosal dissection for gastric mucosal tumor and subepithelial tumor in Kaohsiung Medical University Hospital.

**Results:** Totally, 35 patients with gastric subepithelial tumor and 41 patients with gastric mucosal tumor received endoscopic submucosal dissection are enrolled. Among 35 patients with subepithelial tumor, 32 (91.4%) patients achieved curative treatment. 1 patient received emergent operation and 2 patients received salvage operation to complete tumor resection. 8 patients (22.9%) occurred perforation and no delay bleeding was found. Among 41 patients with mucosal neoplasm, 30 (71.4%) patients achieved curative treatment. 2 patients received emergent operation and 9 patients received salvage operation to complete tumor resection. 9 patients (21.9%) occurred complication, 6 patients occurred delay bleeding and 3 patients had perforation.

**Conclusions:** Comparing ESD between gastric mucosal tumor and subepithelial tumor, ESD had similar efficiency in curative treatment. However, ESD in subepethelial tumor encountered higher perforation and lesser delay bleeding.

## Introduction

Gastric tumor can be divided into epithelial tumor and subepithelial tumor based on its origin. 90% of gastric tumors including adenoma and adenocarcinoma are originated from epithelium 1. If these epithelial tumors are diagnosed as limited to mucosa, endoscopic mucosa resection skills such as EMR and ESD could be reasonable choices to complete tumor local resection with organ preservation. In particular, ESD has a higher en-block resection rate which makes it as a treatment for early gastric cancer 2, 3. Although subepithelial tumor (SET) responds to 10% of gastric tumor, it still has potential malignant and indication of resection exited as an important issue to get pathologic examination and tumor resection 4.

ESD was initially developed since 1990 for mucosal cancer resection, and recent reports proved its curative resection rate reached 95% 5. In the past decade, this skill has also extended its application to subepithelial tumor resection 6, 7. Until now, the indication of ESD for gastric SET was controversial and efficacy was different within previous reports. This retrospective study is design to analyze, compare the efficacy and complication of ESD between epithelial tumor and subepithelial tumor in single center.

## Material and Methods

### Patients

In Kaohsiung Medical University Hospital, we started ESD for gastric tumor treatment since 2007 and extended its application to SET resection since 2011. We retrospectively analyzed all of the cases of mucosal tumor and subepethelial tumor received ESD between January 2007 and December 2016. We identified 76 patients who underwent ESD for gastric tumor, 35 patients SET and 41 mucosal neoplasms respectively. The indications for gastric mucosal tumor ESD were: differentiated intramucosal cancer without ulceration, irrespective of the tumor size; differentiated intramucosal cancer with ulceration, size less than 30mm; differentiated cancer with minute submucosal invasion, size less than 30mm; undifferentiated intramucosal cancer without ulceration, size less than 20mm. The indications for gastric subepithelial tumor ESD were EUS or CT proved intraluminal growing of gastric subepthelial tumor.

### Endoscopic Submucosal dissection

#### Anesthesia

All ESD procedure performed under general anesthesia via endotracheal intubation for air-way protection and vital signs were monitored closely during the procedure.

#### Instruments

Both tip-covered type knife (IT knife, KD-610L; Olympus) and tip-uncovered type (precut needle knife, CD-1L, Dualknife KD-650L, Olympus) were used in endoscopic submucosal dissection. Coagrasper (FD-410LR, Olympus) was used for intra-operation bleeding. Electrosurgical unit used ESG-100 (Olympus). A single channel endoscope (GIF-Q260, GIF-Q260J, Olympus) with hood was used.

#### ESD procedure

Marking was done around 2mm outside the margin of target lesion with needle knife or Dual knife (Force coagulation-1, 20 W). An initial cut was made with a tip-uncovered knife outside the marking after injecting a glycerol solution of epinephrine (1:100,000) and diluted indigo carmine into the submucosal layer. Injection volume varied according to lesion size, and injections were repeated during the procedure as needed. Mucosal circumferential cutting was performed with IT knife (pulse slow cutting 25 W). Submucosal dissection was performed with IT knife or tip-uncovered type knife (force coagulation-2, 40 W). Intra-operation bleeding was managed with coagrasper (soft coagulation, 70 W).

### Histopathological evaluation

The resected mucosal tumor were flattened and fixed at their periphery by thin needles onto a plate of wood, then fixed in a formalin solution. The margins of the tumor, depth, histological type, size, macroscopic appearance and lymphovascular infiltration were assessed pathologically with hematoxylin-eosin staining. Histological classification was microscopically carried out according to the revised Vienna classification of gastrointestinal epithelial neoplasia.

The removed SET were analyzed with hematoxylin-eosin staning and immunohistochemical staining with CD117, CD-34, smooth muscle actin (SMA) and S-100.

### Strategy of follow up

Follow-up endoscopy was performed 6, and 12 months after treatment to assess the completeness of resection in the first year; thereafter, it was examined annually to diagnose local recurrence. Biopsy was repeated for histopathologic evaluation when tumor recurrence was suspected.

### Statistical analysis

The statistical analyses are performed using SPSS software (SPSS, Version 20; Chicago, IL, United States). Quantitative data are tabulated as mean ± SD. Prevalence or positive rates of categorical variables are expressed as a percentage (%). Parametric data are compared using the Student's t-test, while non-parametric data are compared using Chi-Square test or Mann-Whitney U test. A value of P < 0.05 is considered to be statistically significant in all the analysis.

## Results

### Characteristics of patients with SET and mucosal tumor (Table 1)

**Figure 1:**
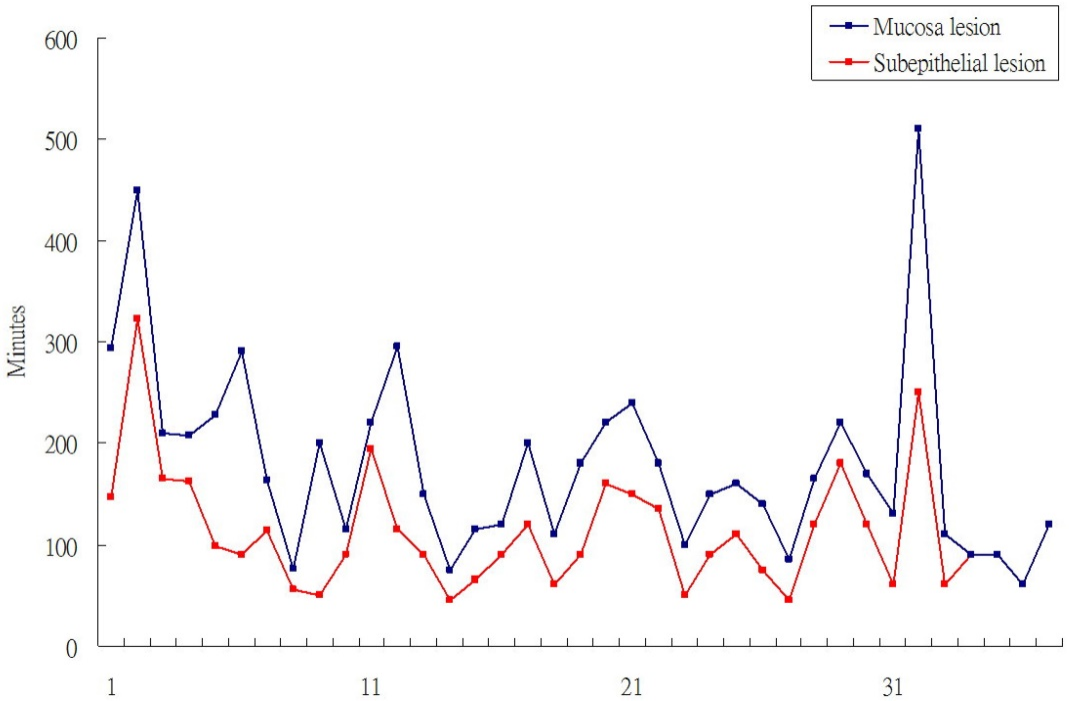
Procedure time of epithethial tumor ESD and SET ESD.

Totally, 35 patient with SET and 41 patients with mucosal tumor received gastric ESD. The location of SET were more often over upper body and mucosal were tend to locate over low body such as antrum/anglaris (P<0.001). Among the 35 patients with SET, 29 patients received 7.5 Mz or 12-Mz endosopic ultrasound (EUS) to evaluate the size and location of SET. It showed 27 SET origined from 4^th^ layer (muscle propria) and two were origined from 3^rd^ layer (submucosal layer). The other 6 SET cases received computer tomography scan before ESD. All of SETs were introlutmial growthing. The mean procedure time of SET ESD was 76.1 minutes and shorter than 111.6 minutes of mucosal ESD (P=0.012). The curative treatment rate were 32/35 (91%), 30/41 (73%) in SETs and epithelial tumor respectively. In the case series of ESD for SET and mucosal tumor also revealed the procedure time of SET ESD were shorter than mucosal tumor ESD (**Figure 1**).

### Complicated and simplified ESD in SET and mucosal tumor

Because of intra-operation perforation, delay bleeding and surgical intervention (including emergent operation and salvage operation for non-curative ESD) would associated with prolong hospital days and complication, all of these situations that occurred would defined as complicated ESD.

**Figure 2:**
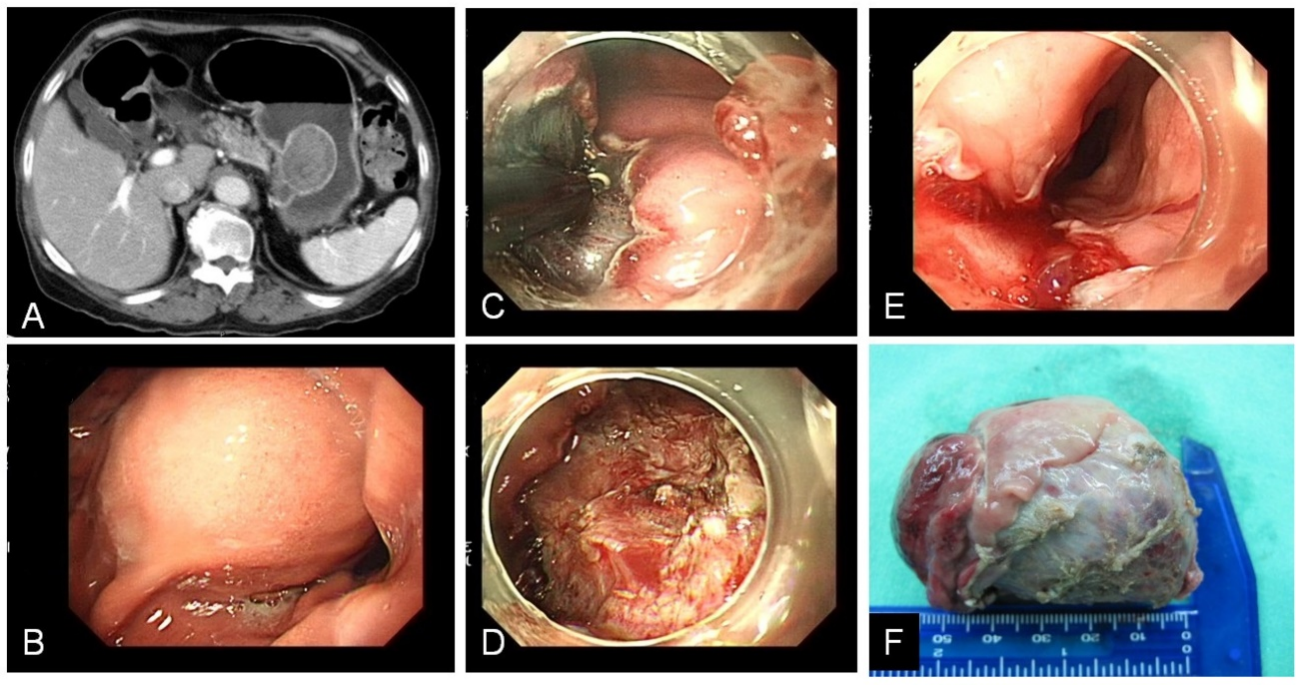
** A.B.** CT and endoscopy showed gastric subepithelial tumor with size more than 3 cm over stomach upper body. **C.** ESD performed with IT-knife. **D.** Post ESD gastric ulcer. **E.** Upper esophagus laceration after specimen extracted out. **F.** Specimen.

In the subepithelial tumor (**Table 2**), 9 patients (25.7%) had encountered complicated ESD. 8 patients occurred intra-operation perforation and 1 patient had salvage operation due to incomplete tumor resection. Among these perforation cases, 2 patients were associated incomplete tumor resection. One received emergent operation for tumor resection with partial gastrectomy, another one use hemoclip to close perforation and received elective surgery for tumor resection 2 days later. The other 6 patients of perforation received conservative medical treatment with endoscopic hemoclip closure and subsequent antibiotics treatment of 5~7 days without surgical intervention. Tumor size bigger than 3 cm wound associated with higher rate of complicated ESD (P=0.027) for not only gastric perforation, but also esophagus laceration as specimen extraction from natural orifice (**Figure 2**). Compared with leiomyoma, GIST wound also associated with higher rate of complicated ESD (P=0.023). In the mucosal tumor group (**Table 3**), 17 patients (41.5%) had encountered complicated ESD. 6 patients occurred delay bleeding, 3 patients had perforation, 9 patients had elective surgery for curative treatment for gastric cancer. Among the 3 cases of perforation, 2 cases received emergent operation for tumor resection and 1 case used endoscopic hemoclip closed perforation and recovery after conservative treatment. Histology with undifferentiated adenocarcinoma would associated with higher rate of complicated ESD (P=0.03).

### Pathologic diagnosis of SET

Among 35 SET, 33 patients (94.3%) got adequate specimen for pathologic diagnosis of SET. 2 patients received operation to achieve pathologic diagnosis because of intra-procedure perforation and incomplete tumor resection. Most SETs were pathologically diagnosed as GIST (45.7%) and leiomyoma (37.1%). Other few SETs were neuroendocrine tumor, schwannoma, lymphangioma, ectopic pancrease (5.7%, 2.8%, 2.8%, 2.8%, respectively) (**Table 4**).

## Discussion

The prevalence of subepithelial gastric lesions was around 0.36% during routine endoscopy examination 8. The differential diagnosis of SETs is not easy and includes non-neoplastic lesions, benign neoplasms, and, potentially, overtly malignant tumors. EUS is a useful tool for evaluation of SETs, but the presumptive EUS and pathological diagnosis matched in only 77-82.9% of cases 9-11. According to the position of the American Gastrointestinal Association Institute, patients with SETs < 3 cm can be followed up with periodic endoscopy or endoscopic ultrasonography (EUS) 12. However, this approach involves issues related to patient compliance, cost-effectiveness, and the risk associated with repeated endoscopic procedures. Therefore, histologic examination is necessary for accurate diagnosis. However, standard endoscopic forceps biopsies, jumbo biopsy and EUS-assisted sampling had reported disappointing result 13, 14.

ESD was innovative method in management of superficial gastrointestinal neoplasm and this technique has become popular in Japan since 2000 15, it has been established as a standard therapeutic method for early gastric epithelial tumor. It may also be considered as a promising minimal invasive technology to treat gastric SETs 16-18.

Overall, the efficiency of ESD in epithelial tumor achieved 95% of curative rate with similar to surgical gastrectomy in previous report 19. However, the present study only had curative rate of 70% in epithelial tumor. It may be related to that the initial epithelial tumor ESD were still within the learning period. Besides, some epithelial cases were undifferential adenocarcinoma (extended criteria of gastric ESD) and it would be more challenge to endoscopic submucosal dissection 20, 21. Our hosptial is low volume hospital of ESD and only 76 ESD procedures were done in past 10 years. The experience of ESD would also influence the success rate and management of adverse event. On the other hand, the curative rate of SET ESD was 90% in the present study. It is not only for treatment purposes but also to make an accurate histological diagnosis.

Although the basic skills of ESD in dealing of mucosal tumor and SET were similar, the target is different in the course of ESD respectively. In epithelial tumor ESD, the dissection is away from muscle layer, but SET ESD has to deal with muscle layer. Most gastric SET origin from 4th layer (muscular propria layer) of gastric wall and submucosa dissection course would have large chance to contact the muscular layer. In present study, the perforation rate was obviously higher in SETs group than epithelial tumor. Oppositely, delay bleeding was higher in epithelial tumor than SET. This may be because most epithelial tumor were larger than 3cm and associated with bigger post ESD artificial gastric ulcer. In the past decade, several novel endoscopic devices promise endoscopic full thickness resection. Over-the-scope clip was showed safety and efficacy in resection SET less than 10 mm 22, 23. Endoscopic suturing system (such as Apollo Overstitch) had applied in larger gastrointestinal wall defect closure and varied endosurgery procedure 24. All of these advance equiement make gastric SET endoscopic section easier.

Report from Choi et al hinted that trainee would need to perform 20±40 procedures to be able to use the technique safely and effectively 25. Others report that experience of at least 30 cases is required for a beginner to gain early proficiency in this technique 26. Another study by N. Kakushima et al was not able to demonstrate an optimal number of cases required to gain adequate experience 27. Besides, it suggested that a beginner could begin to treat lesions in the lower part of the stomach to gain the technique of ESD. There was no report to discussing the SET ESD learning cure. The present study showed SET ESD had shorter the procedure time and higher curative rate. So, the learning cure may be similar to epithelial ESD. However, higher potential of perforation was the important issue in the beginning of SET ESD.

Some limitations exist in the present study. First, this is single center, single operator study. There was no experience ESD operator to supervise the ESD procedure and the learning cure of ESD procedure may be different with other institute. Second, the case numbers were small both in epithelial tumor and SET and most of these case were in initial period of developing ESD. The success rate tends to lower and complication tends to higher. Third, there were not including other endoscopic skill such full-layer resection or STER in the management of SET in this study. Untill now, there still have debates in the endoscopic resection of gastrointestinal stromal tumor eventhough even though advance of endoscopic resection modality 6, 28. Further large scale studies and loger follow-up would warrant clarifying the rule of endoscopic resection in gastric GIST.

## Conclusion

ESD is considered to be a therapeutic technique with a higher radical cure for upper gastrointestine neoplasm treatment, but it may result in a risk of complication. This highly technical procedure needs a high level of expertise and experience to correctly carry out the submucosal dissection and to promptly control any procedure-related complications. Compare with mucosal tumor ESD, SET ESD had high curative rate with proper histology diagnosis. However, higher perforation rate would be important issue in dealing SET ESD in the initial cases. Endoscopic closing skill would be essential for gastric SET ESD.

## Figures and Tables

**Table 1:** Character of gastric ESD

	Gastric subepithelial tumor (n=35)	Gastric mucosa tumor (n=41)	*P* value
Sex (M/F)	12/23	26/15	0.011
Age	62.1 (40~84)	69.1 (29~90)	0.018
**Location**			
Upper	19	2	0.000
Middle	12	1	
Lower	4	38	
Procedure time	76.1 (20~260)	111.6 (45~322)	0.012
Curative treatment	32	30	0.072
**Complication**			
Delay bleeding	0	6	0.016
Perforation	8	3	
**Surgical intervention**			
Emergent op.	1	2	0.110
Salvage op.	2	9	
Hospitalization days	8.4 (3~22)	10.6 (5~22)	0.041

**Table 2:** Comparison of complicated ESD/simplified ESD in gastric subepithelial tumor

	Complicated ESD (n=9)	Simplified ESD (n=26)	*P* value
Mean age	64.3 (40~81)	61.3 (40~84)	0.505
Gender (M/F)	4/5	8/18	0.685
**Location**			
Upper	4	15	0.748
Middle	4	8	
Lower	1	3	
**Size**			
> 3 cm	4	2	0.027
< 3 cm	5	24	
**Pathologic diagnosis**			
GIST	6	10	0.023
Leiomyoma	0	13	
Other	3*	3**	

Complicated ESD: perforation, delay bleeding, surgical intervention, Simplified ESD: curative ESD without complication. *Adenocarcinoma 1, Neuroendocrine tumor 1, schwannoma 1; **Ectopic pancreas 1, Neuroendocrine tumor 1, lymphangioma 1.

**Table 3:** Comparison of complicated ESD/simplified ESD in gastric mucosa tumor

	Complicated ESD (n=17)	Simplified ESD (n=24)	*P* value
Mean age	70.3 (45~90)	68.3 (29~88)	0.657
Gender (M/F)	7/10	19/5	0.013
**Location**			
Upper	1	1	0.464
Middle	1	0	
Lower	15	23	
**Size**			
> 3 cm	13	22	0.094
≤ 3 cm	5	2	
**Pathologic diagnosis**			
Differentiated	11	24	0.03
Undiffirrentiated	6	0	

Complicated ESD: perforation, delay bleeding, surgical intervention, Simplified ESD: curative ESD without complication.

**Table 4:** Pathologic character of subepithelial tumor (n=35)

	Case number (%)
**Gastrointestinal stromal tumor**	
High-risk GIST	5 (14.3)
Low-risk GIST	11 (31.4)
**Leiomyoma**	13 (37.1)
**Neuroendocrine tumor**	2 (5.7)
**Schwannoma**	1 (2.8)
**Lymphangioma**	1 (2.8)
**Ectopic pancrease**	1 (2.8)
**Other**	1 (2.8)
